# Recent Advances in Nanotechnology-Based Diagnosis and Treatments of Human Osteosarcoma

**DOI:** 10.3390/bios11020055

**Published:** 2021-02-20

**Authors:** Mahmood Barani, Mahwash Mukhtar, Abbas Rahdar, Saman Sargazi, Sadanand Pandey, Misook Kang

**Affiliations:** 1 Department of Chemistry, Shahid Bahonar University of Kerman, Kerman 76169-14111, Iran; mahmoodbarani7@gmail.com; 2 Faculty of Pharmacy, Institute of Pharmaceutical Technology and Regulatory Affairs, University of Szeged, 6720 Szeged, Hungary; mukhtar.mahwash@pharm.u-szeged.hu; 3 Department of Physics, Faculty of Science, University of Zabol, Zabol 538-98615, Iran; 4 Cellular and Molecule Research Center, Resistant Tuberculosis Institute, Zahedan University of Medical Sciences, Zahedan 98167-43463, Iran; sgz.biomed@gmail.com; 5 Particulate Matter Research Center, Research Institute of Industrial Science & Technology (RIST), 187-12, Geumho-ro, Gwangyang-si 57801, Korea; 6 Department of Chemistry, College of Natural Science, Yeungnam University, 280 Daehak-Ro, Gyeongsan 38541, Korea; mskang@ynu.ac.kr

**Keywords:** nanotechnology, bone diseases, drug delivery, nanocarriers, osteosarcoma, tumor-targeted

## Abstract

Osteosarcoma (OSA) is a type of bone cancer that begins in the cells that form bones.OSA is a rare mesenchymal bone neoplasm derived from mesenchymal stem cells. Genome disorganization, chromosomal modifications, deregulation of tumor suppressor genes, and DNA repair defects are the factors most responsible for OSA development. Despite significant advances in the diagnosing and treatment of OSA, patients’ overall survival has not improved within the last twenty years. Lately, advances in modern nanotechnology have spurred development in OSA management and offered several advantages to overcome the drawbacks of conventional therapies. This technology has allowed the practical design of nanoscale devices combined with numerous functional molecules, including tumor-specific ligands, antibodies, anti-cancer drugs, and imaging probes. Thanks to their small sizes, desirable drug encapsulation efficiency, and good bioavailability, functionalized nanomaterials have found wide-spread applications for combating OSA progression. This review invokes the possible utility of engineered nanomaterials in OSA diagnosis and treatment, motivating the researchers to seek new strategies for tackling the challenges associated with it.

## 1. Introduction

Osteosarcoma (OSA) is the most common primary metastatic bone cancer in children, young adults, and sometimes in elderlies [1]. Generally, OSA occurs in the proximal tibia, distal femur, proximal humerus, around the knee, and axial skeleton [2,3]. Although the axial skeleton is rarely affected, the aggressive axial skeleton of OSA has been associated with substantially high morbidity compared with other primary tumors within the appendicular skeleton [4,5]. The annual mortality rate of OSA was estimated to be 4.4 per million for individuals <25 years old and 3.1 per million for all ages [6]. Genome disorganization, aneuploidy with chromosomal modifications, deregulation of tumor suppressor genes, and DNA repair defects are the most common characteristics of OSA [7].

At the time of diagnosis, a minority of patients present with metastatic OSA, mainly involving the lungs [3]. Fortunately, an average of 35% of patients with localized OSA will encounter distant recurrence [8]. Studies have shown that patients living in less affluent communities experienced a higher risk of metastatic OSA at the time of diagnosis [9]. Clinicians routinely confirm OSA by the appearance of mixed radiodense and lytic lesions of the metaphyseal bone [10]. Different imaging techniques, including X-ray computed tomography (CT), magnetic resonance imaging (MRI), and positron emission tomography (PET), are widely used for detecting primary and secondary OSA tumors [10,11]. Among these techniques, CT is mostly preferred for skeletal system diseases, since MRI is not sensitive to calcium-enriched bone tissues, and PET scanning has low spatial resolution [12]. In conventional CT, either CT contrast agents or bones generate a similar attenuation of X-ray. Therefore, it is hard to differentiate bones from the surrounding OSA site accumulated with contrast agents [13]. This minimizes the efficacy of CT as the recommended diagnostic approach for OSA [14]. Current strategies of treating OSA patients include preoperative chemotherapy, complete surgical resection combined with a high-dose chemotherapy regimen [15,16]. The success rate of surgical resection against localized OSA is stated to be less than 20%, although when accompanied by chemotherapy, it increases dramatically to about 70% [17]. Chemotherapy has become the patients’ choice for OSA treatment; however, systematic chemotherapeutics induce considerable cardiac and nephron-toxicity [18]. Thus, the use of conventional chemotherapeutics for treating OSA patients is limited by their unfavorable side effects [19]. In addition, poor response to chemotherapeutic regimens might occur, due to the heterogeneity and the genomic complexity of OSA [20].

Nanotechnology is a burgeoning research field that has offered groundbreaking solutions for the diagnosis and treatment of OSA [21]. In this regard, a wide range of nanomaterials has been designed for the targeted treatment of OSA with the least cytotoxicity towards normal human cells [22,23]. As engineered nanomaterials, nanoparticles (NPs) have wide-spread applications in OSA diagnosis and treatment [24,25]. This is primarily due to their specialized structure, desirable efficiency of drug encapsulation, and good bioavailability [26,27,28]. As promising nanocarriers, NPs can deliver various chemicals, drugs, small molecules, peptides, nucleic acids, and even vaccines to the target locations [29,30,31].

Previous studies have indicated that NPs enhance the delivery of chemotherapeutic drugs to OSA cells overexpressing specific antigens or surface receptors [21,32]. PEGylated gold NPs modified with doxorubicin were also more effective than doxorubicin alone for OSA treatment [33]. Newly synthesized NPs loaded with multiple anti-cancer drugs have shown a great advantage in systemic OSA therapy [34]. In addition, NPs possess excellent spectral CT performance have emerged as alternative CT contrasting agents for OSA diagnosis [14,25]. Other nanomaterial-based delivery systems, such as metal nanocages [35], nanocomposites [36], nanocapsules [37], nanoliposomes [38], nanohydrogels [39], and nanomicelles [40], have also provided several advantages over routine therapies for OSA.

Despite significant advances in the diagnosis and treatment of OSA, the overall survival of patients has been stagnant for over two decades [10]. Therefore, recent studies have increasingly focused on improving therapeutic strategies for enhancing the diagnostic accuracy of OSA and combating its progression [41,42]. In this context, following our group’s efforts to synthesize nanomaterials and to investigate their potential bio-applications [43,44,45,46,47], we reviewed different nanomaterials applied for OSA Management.

## 2. Diagnosis of Human Osteosarcoma

### 2.1. Current Approaches for Diagnosis of OSA

Clinicians to identify OSA use several examinations. Some examinations are often conducted to learn whether cancer has spread from, where it originated to another part of the body or not, known as metastasis (metastases is the plural). As cancer cells break away from the main tumor and join the bloodstream or lymphatic system, metastases most generally grow. Fluids are transported across the body by these systems. This means that the cancer cells can travel far from the original tumor and form new tumors when they settle and grow in a different part of the body. When cancer cells from the main tumor, usually in the abdomen or abdominal cavity, break off and expand in surrounding areas, such as the liver, lungs, or bones, metastases may often also develop. Imaging tests use X-rays, magnetic fields, or radioactive substances to create pictures of the inside of the body. Imaging tests are performed for a number of reasons, such as: (i) to help determine whether cancer may be a suspicious area; (ii) to help determine whether cancer may have started in another part of the body; (iii) to find out how far cancer has spread; (iv) to help determine whether treatment is working; and (v) to look for signs that cancer may have returned [48,49]. Today, the most trusted OSA diagnostic tools are imaging tests. The most widely used tool for OSA diagnosis is bone X-ray, chest X-ray, computed tomography (CT) scan, MRI scan, positron emission tomography (PET) scan or PET-CT scan, bone scan, and biopsy which include core needle biopsy and surgical (open) biopsy [50,51,52,53]. For osteosarcoma, the guaranteed option for a specialist to determine whether a body region has cancer is the biopsy method. In this approach, a small sample of tissue taken for examination in a laboratory. A bone scanning process with a radioactive tracer may also be done enough to see through the bones [54]. An MRI method can use with magnetic fields to achieve precise pictures of the tissue and determine the size of the tumor. On the other hand, using X-rays obtained from various angles, a CT scan takes photographs of the inside of the body. These images can be combined by a computer into a detailed, 3D image showing any anomalies or cancers [55,56,57].

Existing approaches to OSA diagnosis such as CT, X-ray, and MRI are limited by the signal intensity and do not detect a small mass of tumors. Detection of tumors largely depends on the visual resolution of the imaging method. Tiny tumors below 1 mm^3^ are unlikely to be identified by CT and MRI [58,59,60]. Scientists have investigated nano-agents for contrast-enhancing imaging methodologies in past years. Due to their targeting capacity and tumor aggregation, functional nanostructures can improve X-ray contrast and detection sensitivity.

### 2.2. Nanomaterials for Diagnosis of Human Osteosarcoma

**Figure 1:**
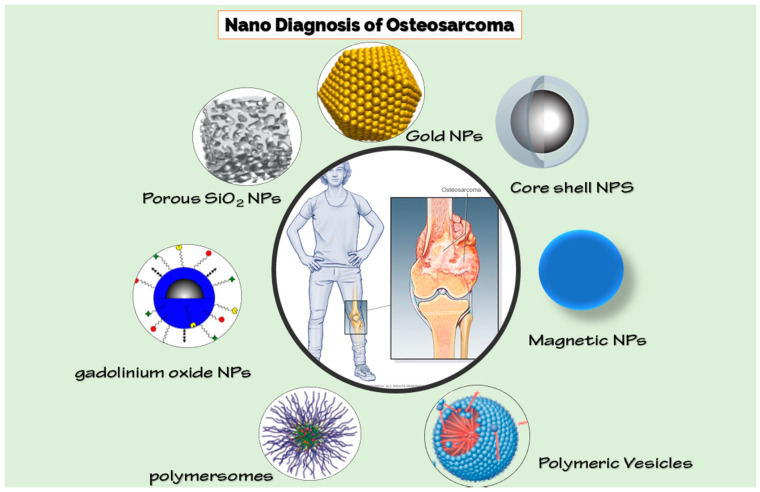
Different nanoparticles for diagnosis of osteosarcoma (OSA).

The nanotechnology approach can improve the diagnostic sensitivity of OSA. Thousands of nanocarriers have reached the clinic, and there are hundreds of nanomaterials proposals being tested by the Food and Drug Administration (FDA) [61,62]. The small size of nanoparticles enables them to overcome biological barriers and reach greater therapeutic effectiveness [63,64,65]. Nanotechnology has combined with the above-mentioned imaging approaches for targeted imaging and can provide a clinical need for high sensitivity and specificity (Figure 1). We have studied the latest state widely of the art of nanomaterials for OSA diagnosis in the following paragraphs.

#### 2.2.1. Single-Photon Emission Computed Tomography (SPECT)/CT Imaging

SPECT has been a cornerstone of the science of nuclear medicine. More recently, in many clinical cases, the combination of the functional imaging available with SPECT and the anatomical imaging of CT has gained more acceptance and been proved useful. SPECT/CT imaging has shown outstanding penetration capacity and is more applicable for deep tissue imaging and especially for OSA imaging [66,67]. A perfect option for the therapeutic management and evaluation of malignant osteolysis could be the imaging and therapy role of versatile nanomaterials [68]. Different forms of nanomedicines based on albumin have been investigated for cancer therapy, guided imaging, and biosensors [69,70,71]. For example, an alternative to therapeutic navigation and monitoring of malignant osteolysis could be to leverage the imaging and therapy role of flexible nanomedicine. Chen et al. reported the development of albumin-based gadolinium oxide nanoparticles loaded with doxorubicin and conjugated with bone-seeking alendronate for targeted delivery and therapeutic monitoring [68]. After radio labeling with ^125^I and SPECT imaging, the authors observed a good distribution of NPs in the body. SPECT imaging also showed the enhanced bone tumor accumulation and prolonged retention of NPs in bone cancer. On the other hand, CT imaging and pathological examination showed that the combination therapy in this study was effective. The finding of this study indicated that albumin-based nanomaterials would provide a system for bone imaging and evaluation.

Developing alternative medical strategies that allow real-time monitoring of drugs and also imaging of tissue is an important nanomedicine approach. A high level of control is provided by real-time magnetic resonance (MR) guidance of laser-induced thermal therapy (LITT). Therefore, this method allows a minimally invasive alternative for resistant focal metastatic intracranial tumors to be killed and treated [72]. In this light, Zhou et al. demonstrated an integrated system for dual diagnosis and treatment of bone tumors. The platform was based on bone-responsive polymeric vesicles with exceptional SPECT/CT imaging ability and good antitumor efficiency [73]. The polymer vesicle is self-assembled from poly(ε-caprolactone)_67_-b-poly((L-glutamic acid)_6_-stat-(L-glutamic acid-alendronic acid)_16_) (PCL_67_-b-P(Glu_6_-stat-(Glu-ADA)_16_)) in water and without a co-solvent. A combination of SPECT/CT and NPs perfectly monitored the distribution of the drug in the bone cancer of in vivo model (rabbits). The study clearly shows the ability of polymer vesicles for simultaneous imaging and successful treatment of malignant bone tumors, offering an optimistic approach for imaging-guided cancer treatment.

In another study, Lu et al. reported enhanced OSA killing and CT imaging using ultrahigh drug loading and NIR-responsive bismuth sulfide@mesoporous silica NPs. Here, authors have prepared a core-shell of bismuth sulfide NPs and mesoporous silica (Bi_2_ S_3_ @MSN NPs) and then attached covalently to arginine-glycine-aspartic acid (RGD) peptide (c(RGDyC)) [25]. The nanoplatform had a perfect sensitivity for OSA and accumulated in cancer cells (10-fold more than peri-tumoral tissue) for better monitoring with CT imaging.

The current positive CT contrast agents (CTCAs) provide a good CT density value (CT-DV) but accurate diagnosis of some diseases such as OSA is limited in this approach [74]. To solve this problem, Meng et al. developed an innovative strategy based on negative CT contrast agents (NCTCAs) to reduce the CT-DV of OSA [74]. They synthesized ammonia borane loaded-hollow mesoporous silica NPs modified with PEG for satisfactory diagnosis of OSA. By reacting to the acidic medium of OSA, nanostructures can generate in situ H_2_ in OSA regions. This result showed a nearly 20-fold reduction in CT density in OSA.

Recently, a progressive type of CT called gemstone spectral CT (GSCT), has obtained great attention. GSCT has a high capacity for material decomposition and monochromatic images to overcome the drawbacks of traditional CT [75,76]. Jin et al. prepared lutecium (Lu)-based up-conversion nanoparticles (UCNPs, PEG–NaLuF_4_: Yb/Er). Lu-based UCNPs showed higher spectral CT performance than iohexol (contrast agent) and can be a better spectral CT contrast agent for the diagnosis of OSA [14]. In vitro and in vivo GSCT demonstrate nanoplatform experiments can provide greater diagnostic elucidation and separate the OSA from the surrounding bones. Owing to the various X-ray amplification properties of UCNPs and iohexol under different energy, iohexol failed to distinguish between cancer bone and healthy bone. The findings thus indicate that the excellent biocompatibility of Lu-based UCNPs has great potential for further clinical diagnosis of skeletal system diseases.

#### 2.2.2. Fluorescence Imaging

Fluorescence imaging is a form of a non-invasive imaging tool that can help to visualize biological processes in a living organism. Images can be created using a variety of techniques, including microscopy, spectroscopy, and imaging probes. The fluorescence imaging approach measures emitted photons by laser-excited fluorescent probes. NPs are usually attached to fluorescent dyes to imagine bone tissues by fluorescence imaging [77,78,79]. A significant prognostic factor for bone tumor growth is lymph sarcoma [80]. Therefore, it is important to develop novel probes for non-invasive and early stages detection of metastatic lymph nodes (MLNs). To address this issue, Yin et al. developed a novel matrix metalloproteinase-2 (MMP-2)-activatable probe constructed with a near-infrared dye (Cy5), a quencher (QSY21), and a tumor-targeting peptide cRGD covalently linked through a radionuclide (^125^I)-labeled peptide substrate for accurate detection of MLNs [81]. The probe produced MMP-2 concentration-dependent fluorescence near-infrared (NIR) upon vicinity with activated MMP-2. The fluorescence radiation provided sensitive and precise imaging of MLNs through optical and SPECT imaging techniques. Zhou et al. designed and synthesized the two homologous forms of fluorescent probes CH1055-PEG-PT and CH1055-PEG-Affibody that show extremely promising results for targeting imaging of OSA and its lung organ metastasis, respectively. it’s found that the close to NIR-II imaging quality of CH1055-PEG-PT is way superior thereto of CT for the first in vivo 143B tumor imaging, and this probe-guided surgery for accurate surgery of 143B tumor [82].

#### 2.2.3. Magnetic Resonance Imaging (MRI)

**Figure 2:**
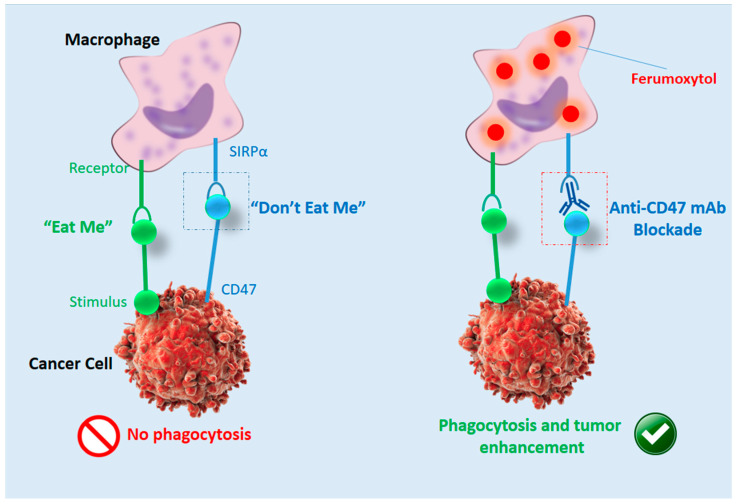
Schematic illustration of ferumoxytol-MRI as an imaging approach for CD47 immunotherapy, reproduced from [86].

MRI is a technology for non-invasive imaging that generates accurate anatomical images in three dimensions (3D). It is also used to track the identification, diagnosis, and treatment of diseases. Due to the high sensitivity of MRI to the reflection of fluid in the body, it provides image data and physiological information simultaneously. Generally, MRI is used in conjunction with contrast agents [83,84,85]. In order to increase the speed at which protons realign with the magnetic field, contrast agents (often containing the element Gadolinium) can be given to a patient intravenously before or during the MRI. The quicker the realignment of the protons, the brighter the picture. Mohanty et al. claimed that in OSA, ferumoxytol NPs can increase MRI and monitor macrophage reaction to CD47 mAb (Figure 2) [86]. Their result showed that tumor-associated macrophages (TAMs) in sarcomas are triggered by CD47 monoclonal antibodies (mAbs) and can kill cancer cells.

As new MRI contrast agents were developed by Pourtau et al. for bone metastasis imaging, multi-functional maghemite NP-encapsulated polymersomes attached to an antibody directed against human endothelial receptor 2 [87]. MRI displayed targeting and improved retention of antibody-attached polymersomes at the tumor tissue after administration in mice carrying bone cancer.

#### 2.2.4. Photo-Acoustic Imaging (PAI)

For the development of successful treatment strategies, detection of different types of tumor remains is important. Ma et al. developed peptide-based probes for photo-acoustic imaging (PAI) and targeted diagnosis of OSA [88]. Using phage display-based monitoring on an OSA cell line (UMR-106), PT6 and PT7 (tumor-specific oligopeptides) were identified. On tissue microarrays, the defined oligopeptides were capable of detecting clinical OSA specimens and pegylated Au nanorods-oligopeptides (PGNR) were explicitly prepared to target UMR-106 cells. More significantly, PAI showed that after systemic administration, both PGNR-PT6 and PGNR-PT7 could specifically attach to subcutaneous UMR-106 xenografts and increase the contrast of OSA pictures by 170% and 230%, respectively in tumor-bearing mice.

#### 2.2.5. Multimodal Imaging

A successful effort to increase the efficiency of diagnosis is the mixture of different imaging techniques [89,90]. A key measure for tumor evaluation and treatment is the invasion stage of tumor-draining lymph nodes (LNs) [91,92]. For more than one imaging technique, multimodal imaging or multiplexed imaging refers to simultaneous signal generation. For example, the use of optical, magnetic, and radioactive reporters to be detected by SPECT, MRI, and PET could be combined. For accurate tumor imaging of OSA, each approach can be combined to create multimodal imaging. In this background, Xu et al. proposed an integrative MRI/NIR/SPECT approach based on 99mTc-labeled gadolinium oxide NPs for improved OSA and tumor-draining lymph node (LN) identification [93]. Nanoplatform with complementary strengths of each modality correctly located OSA tumors. They showed that nanoprobe could be applied in an OSA model with perfect resolution and good sensitivity imaging of lymphatic drainage. In addition, in clinical practice, the nanoprobe can increase the efficacy of the system for nodal resection and tumor staging.

Wang et al. developed a practical dual-modality MR/CT probe for in vivo imaging of OSA [42]. The protein-directed synthesis approach provided an effective alternative to the chemistry-based method. Bovine serum albumin (BSA) is attached to gadolinium NPs (GdNPs) and then iodinated using the chloramine-T procedure. The iodinated BSA-GdNPs (I-BSA-GdNPs) showed a strong coefficient of X-ray attenuation and great drive for MRI. The I-BSA-GdNPs were intravenously injected into orthotopic OSA-bearing rats. Aggregation and retention of NPs in tumors enabled dual-modality and non-invasive imaging. The dual-model, long-circulating I-BSA-GdNPsnanoprobe scan be applied for image-guided surgery and drug delivery application.

## 3. Nanomaterials for the Treatment of OSA

Advancements in nanotechnology have made it possible to deliver the drug to various diseases including OSA. The enhanced surface area and high stability of nanosystems are responsible for improving the bioavailability and drug release profile. Moreover, the hydrophilic and hydrophobic drugs can be incorporated at the same time for the treatment and diagnosis [94]. Nanoparticulate drug systems mainly include polymeric NPs, dendrimers, micelles, liposomes, carbon-based nanovehicles, and metallic NPs. However, most existing and advanced NPs for OSA are made of various kinds of materials.

### 3.1. Polymeric Nanocarriers

Polymers have always been in the highlight because of biocompatibility and biodegradability. Most of the current research is being focused on the delivery of drugs, peptides, genes, etc. by polymeric vehicles [95]. In OSA, polymeric NPs have been very much in demand for effective targeting. In a current study, nanostructures vehicles were designed with poly (ester amide) to deliver Apatinib (Apa) to enhance the survival rate and inhibit the recurrence of the OSA. Due to its encouraging anticancer activity, Apatinib (Apa), a highly selective VEGFR2 inhibitor, attracts considerable attention, particularly in combination therapy clinical trials. VEGF receptor 2 (VEGFR2) inhibitors targeting tumor angiogenic pathway have been widely used in the clinical cancer treatment. The NPs accumulated at the target site and induced apoptosis to significantly enhance the OSA therapy [96]. Another study focused on the chitosan-based nanocarriers for the treatment of OSA. Poloxamer modified trimethyl chitosan was constructed into NPs and later, encapsulated with methotrexate (MTX) for the accumulation in the cancer cells. Methotrexate (MTX), formerly known as amethopterin, is an immune-system suppressant and chemotherapy agent. It is used to treat cancer, ectopic pregnancy, autoimmune conditions, and surgical abortions. The nanostructures exhibited higher infiltration in the cancer cells cytoplasm by endocytosis as was seen by the fluorescence imaging. Additionally, NPs proved to be highly cytotoxic to the MG-63 cells as compared to free drugs and demonstrated a high apoptosis ratio [97].

Poly (lactide-co-glycolide) (PLGA) NPs were synthesized in another research for the effective delivery of the drug to the OSA cells. Salinomycin was used as an anti-cancer drug. The NPs inhibited the tumor signaling pathway and induced apoptosis by the caspase-3 expression in the OSA MG-63 cells. The NPs sustained the release of salinomycin up to 45 days enabling the appropriate treatment of OSA [98]. The other latest NPs based on bis-triazoledcycopolylactides were synthesized by the click chemistry reaction. Salinomycin was loaded in the NPs with high efficiency. Moreover, the NPs were cytotoxic to MF-63 cells and cancer stem cells, and evoked a higher cellular response than the free drug. The NPs were internalized in the cancer cells with high efficiency (72 h) which was revealed by the internalization fluorescence-based studies. Inserting feature of the NPs was the jellyfish architecture, which was a study of its kind [99]. Alginate is another polymer being studied for drug delivery in cancer. Alginate oligosaccharide displays anti-tumor properties and is explored in OSA. Alginate oligosaccharide NPs were prepared and were numbered as, DP2, DP3, DP4, and DP5 based on the extent of polymerization. The clinical studies on the OSA patients revealed that DP5 was highly cytotoxic when administered orally. The mean tumor volume reduced with the reduction of anti-inflammatory cytokines after 2 years of therapy. The antioxidant property of the NPs was highly promising [100]. PLGA NPs were constructed as a matrix system by the incorporation of hydroxyapatite and coated with doxorubicin. NPs exhibited high tensile strength and showed adherence with the bone cells because of the matrix hydroxyapatite. Moreover, the nanocomposite was found to be highly cytotoxic to the OSA cells [101].

### 3.2. Liposomes

Liposomes are considered as model biomembranes for site-specific delivery. The surfaces of liposomes are decorated with ligands. Moreover, they are comprised of natural cholesterol and non-toxic phospholipids that make them biocompatible for the delivery in the OSA. They are more stable than other nanocarriers and can make use of structural variations strategy in the head, tail and bond of lipid structure [102]. Liposomes with hyaluronic acid coating carrying doxorubicin were synthesized for the delivery in the doxorubicin resistant OSA. Doxorubicin was eliminated by P-glycoprotein efflux pump, hence the drug was conjugated with H_2_S releasing moiety. The liposomes were effectively taken up by the OSA cells by binding to CD44 receptors. The cardiotoxicity was reduced and the toxicity to the cancer cells was enhanced [103].

**Figure 3:**
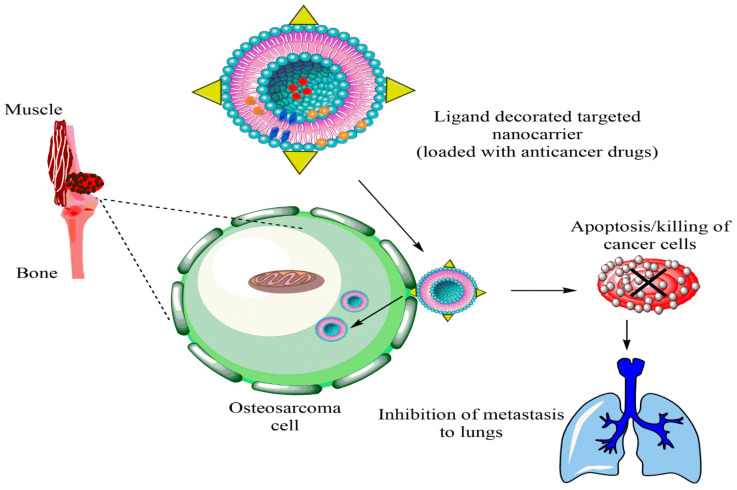
Hypothetical representation of liposome used for multiple targeting strategies in cancer.

In another liposomal formulation, low molecular weight (LMW) heparin was used for the orthotopic OSA. Alendronate was also encapsulated together with anti-tumor doxorubicin. Alendronate is used to treat and prevent osteoporosis. LMV heparin enhanced the blood circulation duration of liposomes. The liposomes demonstrated anti-metastasis property in the OSA model and bone cancer metastasis model [104]. Liposomes with natural plant alkaloid voacamine were developed and the effect of the composition of lipid components was evaluated on the outcome of the treatment. The liposomes were designed for doxorubicin resistant OSA. Phospholipids and cholesterol components resulted in the stable transmembrane difference at pH gradient and accumulated the plant alkaloid within the core. The alkaloid loaded liposomes were more effective than free voacamine for OSA [105]. Surface ligand decorated liposomes had been very popular for the OSA targeted drug delivery. Ephrin alpha 2 receptor (EphA2) has been discovered to be upregulated in the OSA. Doxorubicin loaded liposomes were anchored with YSA peptide to target EphA2 on the OSA cells. The liposomes were PEGylated to improve their circulation in the blood. Designed liposomes increased toxicity 1.9-fold compared to free doxorubicin in the Saos-2 cell model along with high cellular uptake [38]. A hypothetical representation of liposome used for the multiple targeting strategies in cancer is shown in the Figure 3.

New strategies for the use of multiple drugs with synergistic effects for the treatment of pathologies such as OSA have been developed [106]. Dual liposomal-based drug delivery systems are being introduced for OSA. A dual liposomal system was recently developed, simultaneously loaded with gemcitabine (anti-cancer) and clofazimine (anti-inflammatory). The liposomes had higher encapsulation efficiency for both the drugs. The anti-cancer drug was released at a slow rate as compared to anti-inflammatory drug. The co-loaded liposomes had higher caspase-3 activity as compared to other liposomes. Hence, co-loaded liposomes were a promising novel approach for the treatment of OSA [107]. Combinatorial drug-loaded pH-sensitive liposomes were studied for the enhanced treatment of OSA. Doxorubicin (DOX) and ladirubicin were co-encapsulated in liposomes. Liposomes were optimal in size and exhibited biocompatibility with no significant effect on the normal cells. The tumor lesion reduced to half after treatment with the liposomes and therefore can be considered as the promising therapy for malignant OSA [108].

### 3.3. Metallic Nanoparticles

In recent times, metallic NPs showed great advancement in the field of therapeutics and biosensing. Metallic NPs show low cytotoxicity to normal cells and high targeting and localization in the cancer microenvironment. In addition, their size and conformation make them ideal for the targeted drug delivery in OSA [109]. Platinum NPs (PtNPs) exhibit safe and thermally stable, highly effective with good sensing properties and enhanced plasmonic properties [110]. In a similar way, silver NPs (AgNPs) have been associated with the wound healing characteristics and antioxidant properties. AgNPs are also antibacterial and antipathogenic [111].

Titanium dioxide NPs (TiO_2_ NPs) demonstrate biosensing and targeting activity in cancer and are safe with high safety profile and high chemical stability [112]. AgNPs and AuNPs were synthesized to inhibit the human OSA. The synthesis of NPs was performed by green technology. In vitro studies on cells exhibited IC_50_ values of 29.22 ± 0.42 for AgNPs and 32.83 for AuNPs. Mainly, the AgNPs acted as scavengers and sensed the hydrogen peroxide (H_2_O_2_) in the tumor microenvironment [113]. Interestingly, the concept of delivery of NPs synthesized by green technology is gaining interest. Tannins derived from plant extracts reduce the silver ions to the AgNPs. In one such research, AgNPs were prepared from the extract of amangrove plant, *Rhizophora apiculata*. The AgNPs were characterize for the physicochemical properties and evaluated for the cyto toxic effect against OSA cells. The antioxidant properties of AgNPs were the main reason for the reduced viability of the OSAMG-63 cells [114]. The size-dependent activity of AuNPs has been explored in the latest study for the treatment of OSA. AuNPs were synthesized by tris-assisted citrate-based method and size was found to be between 40-60 nm. The AuNPs of 46 nm size enhanced the reactive oxygen species (ROS) induced apoptosis in MG-63 OSA cells. Cell viability was reduced for 46 nm AuNPs and cytotoxicity was reduced with the increase in the size of AuNPs [115].

AuNPs were also exploited in another research, where they were functionalized with PEG. Tat peptide and doxorubicin were conjugated on the surface of the AuNPs to increase the efficacy. Tumor viability assay highlighted that the PEGylated AuNPs were 100% cytotoxic to OSA cells as compared to non-functionalized AuNPs [33]. Similarly, PtNPs encapsulating doxorubicin were evaluated for anti-cancer activity in human U2OS osteosarcoma cells (One of the first developed cell lines is the human osteosarcoma U2OS cell line, which is used in different areas of biomedical research). The PtNPs inhibited OSA cell viability in a dose-dependent manner with upregulation in apoptosis and apoptic gene expression. The PtNPs also promised the increase of oxidative stress induced DNA damage [116]. Surface modified TiO_2_ NPs were developed for the treatment of OSA. Folic acid (FA) was tagged on the surface of metallic NPs due to its affinity for the folate receptors upregulated in cancer. The FA anchored TiO_2_ NPs significantly increased cancer cell apoptosis and exhibited high infiltration in the OSA cells. Additionally, the TiO_2_ NPs also produced ROS and hence facilitated the cell apoptosis and higher expression of caspase-3 [117]. Interestingly, two metallic nanostructures, zinc oxide (ZnO) and cerium oxide NPs, were synthesized by green technology using leaf extract of *Rubia codifolia*. The biological activity was evaluated against MG-63 OSA cells. Apoptosis was found to be very high and cell damage was observed after induction of ROS [118]. ZnO NPs have the ability to kill OSA cells by the upregulation of hypoxia inducible factor 1-alpha proteins for killing OSA. In vivo assay also confirmed the safety of ZnO NPs in the research conducted [119].

### 3.4. Redox Responsive Nanocarriers

A latest trend in the treatment of OSA employs the methods such as stimuli responsiveness. These stimuli can be redox potential, tumor acidity and enzymes which trigger drug release in the tumor microenvironment [120]. Presently, redox sensitive NPs are being developed for targeted delivery in OSA. Based on this idea, redox sensitive liposomes conjugated with hyaluronic acid were synthesized for the drug delivery in the OSA, which used the redox potential of the tumor as stimuli. The liposomes were targeted towards CD-44 receptors to enhance chemotherapy in OSA. Later, the liposomes were stabilized with PEG conjugated with cholesterol. Doxorubicin was used as a model drug for cytoplasmic drug delivery in OSA. PEG-cholesterol conjugated hyaluronic acid(HA) liposomes suppressed the tumor with reduced liver uptake compared to normal liposomes. Hence, CD-44 targeted intracellular drug delivery vehicle proved to be promising [121].

**Figure 4:**
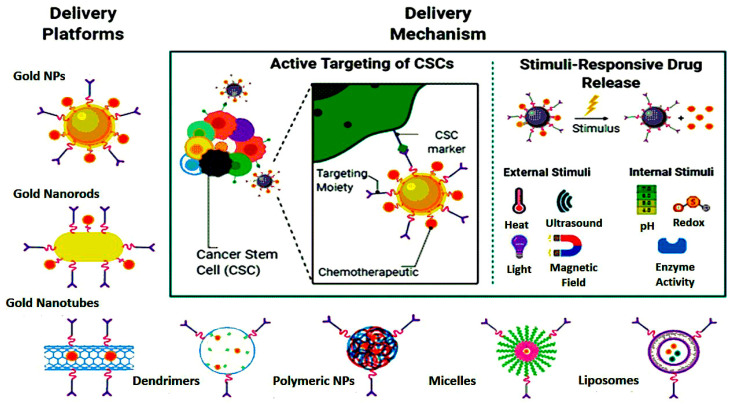
Nanoparticle-mediated targeted drug delivery to cancer stem cells (CSCs), created in Biorender.com.

Additionally, another study by Feng et al. strongly suggests an effective OSA-targeting liposome system functionalized with a dual-targeting polymer redox-cleavable, bone- and cluster of differentiation 44 (CD44). Here, the effect of a tumor-penetrating peptide, internalizing RGD, was clearly shown by Feng and his colleagues [122]. In this case, alendronate (ALN), a bone-targeting moiety, was first conjugated with HA, a ligand for CD44. Via a bioreducible disulfide linker (-SS-), this ALN-HA conjugate was coupled with DSPE-PEG2000-COOH to obtain a functionalized lipid, ALN-HA-SS-L, to be post-inserted into constructed liposomes loaded with DOX. In addition to strong and fast cellular uptake, ALN-HA-SS-L-L/DOX had considerably higher cytotoxicity for human OSA MG-63 cells compared to different reference liposomes. ALN-HA-SS-L-L/DOX displayed impressive tumor growth suppression and extended survival time in the orthotopic OSA nude mouse models. This finding indicates that a successful OSA-targeted therapy can be ALN-HA-SS-L-L/DOX, designed with bone- and CD44-dual-targeting skills and redox sensitivity. Co-administration of internalizing RGD might also improve effectiveness. In another research, a cationic liposomal estrogen linked system was developed for the targeted delivery to OSA. Chotooligosaccharides were covalently attached to liposomes through disulfate linkage and estrogen was anchored via PEG for estrogen receptor targeting. In addition, doxorubicin was embedded in the liposomes for the treatment of OSA. The liposomes released the drug in response to tumoral intracellular glutathione. The tumor targeting was investigated by studying the uptake in MG-63 OSA cells. Furthermore, the chotooligosaccharides grafted estrogen functionalized cationic liposomes exhibited intracellular drug delivery to the estrogen receptor expressed on the OSA cells [123]. Other similar chotooligosaccharides surface coated redox sensitive fusogenic liposomes were studied for OSA. Doxorubicin was loaded in the liposomes for anti-tumor activity. These liposomes were found to be stable with low drug leakage and higher cytotoxic level in the cancer cells. The redox sensitive liposomes had higher cytotoxicity to MG-63 OSA cells than the normal liposomes and had lower cytotoxicity for the LO2 liver cells. Altogether, the liposomes extended survival rate in animals [124]. Some of the nanocarriers based on stimuli responsive drug release are shown in Figure 4. The figure also explains the unique concept of cancer stem cells (CSCs) targeted therapy.

**Figure 5:**
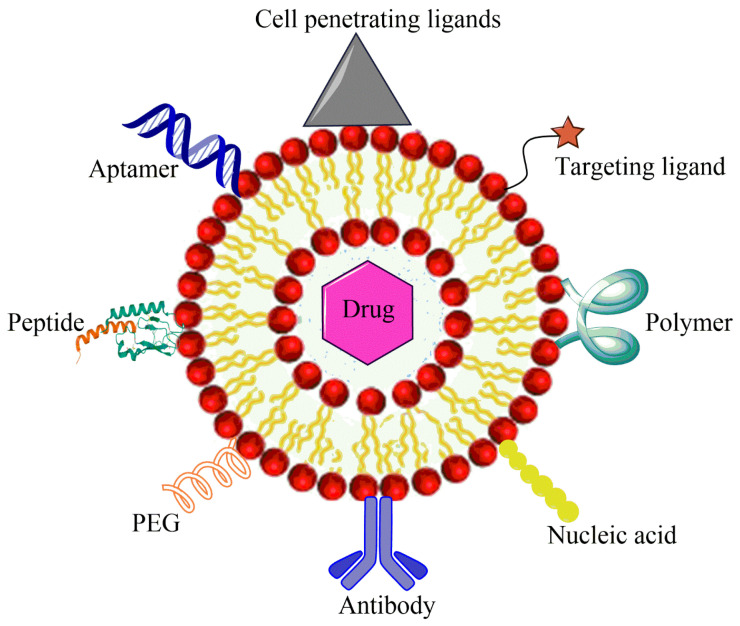
Ligand anchored nanocarrier and its interaction with OSA cell.

The cancer stem cells (CSCs) express the surface markers that can serve as therapeutic targets for the elimination of CSCs. Different nanocarriers can be used for OSA therapy and can be decorated with surface ligands, specific to the CSCs markers and can release the embedded moiety by the stimuli. Figure 5 shows ligand anchored nanocarrier and its interaction with OSA.

### 3.5. Hybrid Nanoparticles

Currently, the combination of various nanomaterials is being developed to create a hybrid structure for enhanced stability and biocompatibility together with improved targeted delivery. A lipid–polymer hybrid nanostructure was synthesized to target CSCs in OSA. Salinomycin was incorporated in the hybrid NPs for its potential activity against CSCs. PLGA and DSPE-PEG along with soybean lecithin were merged together prior to drug loading. Additionally, epidermal growth factor (EGFR) aptamer was conjugated on the surface of hybrid NPs to target the EGFR overexpressed in OSA. The drug was released over 120 h in a sustained pattern. The hybrid NPs exhibited higher cytotoxicity than the un-modified NPs with a notable reduction in the CD133^+^ OSA CSCs in the OSA cell line. Hence, it was a promising strategy for targeted drug delivery to the CSCs [32].

In another research, hybrid NPs comprised of lipid and polymer combinations with a surface decorated CD133 aptamers for the delivery of all-trans retinoic acid to OSA cells. All-tans retinoic acid effectively targets and treats cancer initiating cells; hence, it was encapsulated in the hybrid lipid–polymer NPs. The tumorsphere formation assay and a cytotoxic assay of the hybrid NPs were promising. All-trans retinoic acid was revealed over 144 h and the hybrid NPs were internalized in OSA cells and therefore proved to be the novel approach for the therapy of OSA [125]. In other interesting research, combined metallic and polymeric NPs for treatment of OSA was reported. Copper loaded chitosan NPs were synthesized and were spherical in size. The hybrid NPs had greater internalization than free CuSO_4_. In addition, the NPs exhibited greater mitochondrial ROS and caspase-3 activity [126]. Table 1 lists some nanotherapeutics designed for the OSA.

### 3.6. Mesoporous Silica Nanocarriers

The enormous versatility of mesoporous silica nanoparticles enables a large number of cancer treatment nanotherapeutic systems and several other pathologies to be developed. These materials permit a large number of molecules of a very different nature and scale, in addition to the controlled release of small drugs. The use of mesoporous silica-based NPs have widely increased because of their stability, large surface area with porous structure and biocompatibility. They have tunable properties leading to the high drug encapsulation capacity. As reported, mesoporous silica coated bismuth sulfide NPs eradicated the tumor and released the drug by the near-infrared trigger. The NPs were conjugated with RGD peptide for high targeting efficacy to the OSA. The mesoporous silica NPs activated mitochondrial apoptosis and promoted cell death of OSA [25]. Mesoporous silica NPs have been widely explored for the release of doxorubicin by modulating the functional groups attached to the surface. Amine, sulfonate, PEG, and polyethyleneimine groups were attached separately on the silica NPs. Moreover, the antibody conjugated mesoporous silica NPs was synthesized. Altogether, the functioning of these mesoporous silica NPs were investigated for the cytotoxic activity on the OSA cells. Sulfonate anchored NPs were internalized actively in the presence of serum proteins as compared to the antibody conjugated mesoporous silica NPs, which highlighted that surface charge is of prime importance in the targeted drug delivery to OSA cells [146]. The mesoporous silica NPs were used in the novel nanodevice. The nanodevice comprised of polyacrylic acid (PAA) grafted to mesoporous silica NPs and a targeting ligand (plant Concanavalin A (Con A)) to target the glycan overexpressed in the tumor cells. The nanodevice was 100% cytotoxic to the OSA cells sowing that the nanosystem was highly toxic due to its structure [139].

Recently, cell membrane coating was explored for OSA. Photothermal therapy as the widely used approach was merged with the nanotherpaeutics. Silica NPs were conjugated with the cell membrane originating from the 143B cells to develop the novel targeting strategy. Furthermore, photothermal agent was loaded in the mono-dispersed membrane coated NPs. The silica NPs demonstrated great cytotoxicity because of the membrane coating and photothermal moiety as compared to simple silica shells [147]. In an alternate approach, magnetic core-shell silica NPs were synthesized for the delivery of small interfering RNA (siRNA) to the OSA. Large pore silica NPs were coated with super-paramagnetic nanocrystals and siRNA in the core was protected by tannic acid component, hence serving as pH sensitive system. The external magnetic field accumulated the nanocarriers in the OSA cells [148].

### 3.7. Calcium Phosphates Nanocarriers

Due to its biocompatibility, biodegradability, pH responsive function, and can encapsulate in a variety of drugs in the matrix, calcium phosphate (CaP) was engineered as a drug delivery nanocarrier almost 50 years ago. The CaP nanocarriers for cancer imaging, therapy, and theranostics have been used for loading probes, nucleic acids, anticancer drugs, and photosensitizers. Moreover, they do not release the drug in the physiological plasma condition and release the drugs only in the acidic tumor environment. CaP NPs offer great biocompatibility in cancer therapy. CaP NPs loaded with caffeic acid, chlorogenic acid, or cisplatin were used in the presence of alginate polymer to minimize the burst release of the drugs. The drugs encapsulated in the CaP NPs exhibited anti-cancer activity in the concentration dependent manner [149]. CaP NPs are now being developed as non-viral transfection agents by adjusting the ratio of Ca and P molar ratio. Poly (L-Lysine) was used as a surface additive to optimize the transfection with plasmid DNA encoding a green fluorescent protein in the MC3T3 E cells (pre-osteoblastic). The nanosystem was less cytotoxic than the commercial viral carrier. OSA cells were four times more easily transfectable than pre-osteoblastic cells [150].

In a novel approach, bone substitute material, CaP was used as a scaffold for the resection of bone tumor. The CaPbeads were used for the delivery of cisplatin, doxorubicin and cis-diamminedichloroplatinum (CDDP). Doxorubicin was released continuously for 40 days whereas CDDP was burst released. The beads demonstrated cytotoxicity against MG-63 cells and proved promising for the therapy of OSA [151]. Functionalization of CaP with bioactive agents is a promising strategy in the bone targeted OSA therapy. The R enantiomer of 9-hydroxystearic acid (9R-9-HAS) inhibits tumor proliferation. Hence, 9R-9-HAS was incorporated in the CaP nanocrystals that modulated the cytotoxic effects on the OSA cells. The proliferation was reduced in the tumor cells by the increase of tumor necrotic factor [152]. Similarly, hydroxyapatite (a natural form of calcium apatite) NPs doped with selenium can fill the bone defects caused by tumors. The selenium released from the bone calcium-based structures induced the apoptosis of bone cancer cells by generating ROS. Additionally, the systemic toxicity was educed and tumor formation was inhibited [153]. In a similar, but novel approach, hydroxyapatite NPs were loaded with medronate, a bisphosphonate for targeting the bone cancer, and JQ1 as a small molecule bromodomain inhibitor as a chemotherapeutic. Medronate NPs had a high affinity for the hydroxyapatite. The NPs loaded with both JQ1 and medronate were cytotoxic against OSA cells in the 2-D culture and were completely compatible with the fibroblasts. OSA cells internalized the JQ1 loaded NPs efficiently [154].

### 3.8. Other NPs

Some other nano mediated drug delivery systems have been explored for the delivery of the drug in the treatment of OSA. Other therapeutic options involve the targeting of the surface expressed receptors on the OSA cells. CXCR1 marker is overexpressed on the tumor cells and in OSA and is related to the chemotherapy resistance. CXCR1 targeting peptide was anchored to the magnetic NPs loaded with cisplatin. The NPs inhibited the cancer growth and prevented metastasis of the cancer cells to the pulmonary area [155]. Multi-functional micelles were developed and were loaded with curcumin because of its potential as an antitumor moiety. The micelles were synthesized by using amphiphilic alendronate-HA-octadecanoic acid. The nanomicelles were studied for their efficacy in OSA along with their bone affinity profile. Nanomicelles adhered to the bone because of the composition and released curcumin in a sustained manner. The cytotoxic effect of the nanostructures was pronounced [156]. Polymeric micelles are now being explored for photodynamic therapy by using potential photosensitizers for OSA. Zinc phthalocyanine is a dynamic photosensitizer with excellent photochemical properties. The poor solubility of zinc phthalocyanine was rectified by incorporating it in the poly(ethylene glycol)-pol(2-(methylacryloyl)ethylnicotinate)(PEG-PMAN) coblock micelles. ROS was significantly increased after light irradiation and exhibited 100% cytotoxicity as compared to the free photosensitizer [157].

Similarly, doxorubicin loaded self-assembled micelles were developed from RGD block copolymer poly(ethylene glycol)-block-poly (trimethylene carbonate). The half maximal inhibitory concentration was low as compared to the non RGD nanostructure that highlighted that RGD NPs have high cell targeting ability and anti-tumor effect in OSA [158]. In another similar approach, doxorubicin was loaded in the acid sensitive micelles for the OSA therapy. Hydrophilic D-aspartic acid octapeptide is a very promising micelle corona. Polymeric micelle was stabilized and loaded with the drug by an acid sensitive hydrazine bond. The stability of the polymeric micelles was increased by the increase in the concentration of aminoundecanoic acid to regulate the hydrophilic and hydrophobic ratio. Furthermore, the cytotoxicity was enhanced for the Saos-cells [159]. The polymeric micelles are also being investigated for the anti-cancer drug PENAO (4-(*N*-(*S*-penicillaminylacetyl) amino) which is currently in clinical trials for solid tumors. Direct PENAO polymeric micelles were developed by amidation reaction followed by polymerization with poly(ethylene glycol methyl ether methacrylate) as comonomer and poly(methyl methacrylate) (pMMA) as chain transfer agent, resulting in a coblock polymer. PENAO was readily available to actively target the mitochondria and inhibit cancer. Hence, it can provide a rationale platform for the OSA treatment [160].

OSA cells overexpress HER-2 receptors, thus making HER-2 a target for anti-HER-2 antibody trantuzumab. A nanomaterial structure of graphene oxide (GO) was developed and anchored with anti-HER-2 antibody by covalent bonding. The graphene nanostructure induced cell apoptosis by oxidative stress and leas to the formation of necroptosome. It also elevated the survival rate in animals, thus providing a promising curative therapy for OSA [161]. Alternatively, chitosan NPs were functionalized with GO for delivering siRNA to the OSA Saos-2 and M63 cells. ROS assay demonstrated the biocompatibility of nanoconjugate system and released siRNA in a controlled manner to the tumor site. Expression of inflammatory cytokines was reduced and the cancer cells were killed followed by the uptake in the cells [162].

Another nanosystem is dendrimer, which was reported to inhibit OSA. Dendrimer comprised of amphiphilic block copolymer poly (ethylene glycol)-poly (2-(methylacryloyl)ethylnicotinate)(PEG-PMAN) was synthesized and loaded with zinc phthalocyanine, used as a photosensitizer. The dendrimers elevated the ROS levels upon irradiation with light and killed OSA cells with high effectiveness [157]. Likewise, graphene-based dendrimers were developed to carry magnetic moiety for the delivery of multiple drugs in OSA. DOX and melatonin were coloaded in the branched nanostructures. Studies on Saos-2 and MG-63 osteosarcoma cells exhibited the down regulation of anti-apoptotic components and hence increased cytotoxicity [163]. The PAMAM dendrimers were mounted on the multiwalled carbon nanotubes and explored for the cytotoxicity to the OSA MG63. The nanoconjugate system was stable and biocompatible. The system also decreased the cellular toxicity by 70% which was previously very high for the multiwalled carbon nanotubes (MWCNTs) [164].

Currently, exosomes derived from mesenchymal stem cells are gaining interest in the treatment of OSA. In one such study, DOX was loaded in the exosomes and was analyzed for the in vitro uptake in the MG-63 cells. Exosomes exhibited high infiltration in the MG-63 cells but low uptake in the myocardial H9C2 cells, hence proving to be promising for OSA targeted delivery [165]. Figure 4 highlights some of the nanostructures designed for the drug and gene delivery to OSA.

Recently, new trends are being explored for the treatment of various pathologies including OSA, based on self-assembling peptides. Such peptides can be explored by adjusting their peptide sequence, hence providing an opportunity for the generation of peptide of desired characters. Self-assembly of peptides creates a complex structure of high order for exploration in nanobiotechnological applications [166]. Currently, peptide nanofibrils are gaining interest as they disassemble inside the body and alter or support tissue growth, to make them free of any foreign material [167]. Similarly, ultra-short peptide hydrogels have been found to be efficient in delivering the drug to the cancerous cell. Such peptides perform a dual functions; initiate the growth of new cells and kill the cancerous cells [168].

It is now being studied that changing the peptide molecular properties might affect its interaction with small drugs and influence the release of the drug. The peptides being explored also undergo cytocompatibility studies to affirm their use as a drug delivery tool for biomedical use. The peptides have been found cytocompatible and do not illicit immune response. However, this novel idea still needs extensive research in the field of oncology. The future holds various horizons to be explored for the treatment of OSA.

## 4. Conclusions, Challenges, and Perspectives

Over the past few years, a significant number of targeted nanomaterials have been established for the diagnosis and treatment of malignant bone tumors such as OSA. It is imperative to provide a better understanding of the fundamental concepts involved in the design and application of nanoparticles for diagnosis, treatment, or the combination of imaging and therapeutics in various clinical circumstances, following this remarkable progress in the advancement of nanotherapeutic and imaging methods for cancer detection and treatment. OSA has rapidly metastasizing ability and proves challenging for the treatment rationales. Nanotherapeutics being developed for the OSA include metallic, lipid, polymeric, magnetic and stimuli-sensitive drug delivery systems. Nanotherapeutics improve the safety and compatibility profile in the diseases by minimizing the off-target accumulation. However, tumor biology itself plays a critical role and needs to be studied extensively for the outcomes in the case of nanoparticles therapy. The majority of the nanostructured approaches are in the cellular stages of drug delivery and need to be translated into clinical trials after extensive research. While these targeted NPs showed satisfactory benefits in OSA diagnosis and therapy, there are still difficult problems to solve in the future. For instance, in vivo verification of nanoparticles, and especially subsequent toxic evaluation and bone tissue targeted delivery for either cancer bone metastasis or other bone diseases still require further and extensive experiments to accelerate their potential clinical implementation. Some nano polymeric materials are not very strongly cytotoxic and it can also be expected that they will be offered to humans in the coming years. Nanotechnology is expected to play a pivotal role in future OSA diagnostics. With the development in technology, more powerful diagnostic techniques such as multimodal imaging can be seen in the coming days. Physicists, chemists, engineers, biologists, and clinicians, motivated by the rapid and encouraging developments in nanotechnology, will continue to challenge themselves to design innovative and efficient nanosystems for cancer diagnosis and treatment.

## Figures and Tables

**Table 1:** Nanoparticulate systems developed for the treatment of osteosarcoma.

Nanomaterials	Composition	Loaded Moiety	Outcomes Reported	References
Polymeric NPs	PLGA with surface CD133 aptamers	Salinomycin	Targeted CD133^+^ OSA cell and reduced the progression of osteosarcoma by enhanced infiltration in the cells	[127]
Polymeric NPs	Polydopamine, alendronate	Paclitaxel	Increased accumulation in the tumor cells as compared to other tissues	[128]
Polymeric NPs	PEG-bisphosphonate	Doxorubicin	Internalization by the cancer cells and suppression of tumor growth by cytotoxic effect	[129]
Polymeric NPs	Polylactide coated with pamidronate	Doxorubicin	Malignant bone targeted drug delivery with no cardiac and hematological toxicity, significant anti-tumor activity	[130]
Liposomes	DSPE-mPEG *	Doxorubicin	Thermo and pH sensitive release of drug in the OSA cells	[131]
Liposomes	DSPE-mPEG, cholestrol	Doxorubicin and SiRNA	Dual targeting of the OSA cells surface EphA2 receptors and intracellular JIP1 protein, increased nuclear localization of the liposomes	[132]
Liposomes	TPGS **, phosphatidylcholine, DSPE	Doxorubicin and vitamin E	Concentration dependent toxicity in the OSA cells and high apoptosis	[133]
Liposomes	phosphatidyl ethanolamine	Muramyl tripeptide	Stimulated macrophages to destroy the OSA tumor cells	[134]
Gold NPs	Tannic acid, HAuCl_4_	---	Increased expression of proapoptotic protein Bax in the OSA cells and decreased expression of anti-apoptotic protein Bcl-2	[135]
Metallic NPs	Self-assembly of ferric ions with hyaluronic acid anchorage	Zoledronate	Inhibition of osteoclast activity, generated free radicals killed the OSA cells	[136]
Zinc oxide NPs	Titanium substrate and zinc acetate	Naringin	Reconstruction of large bony defects in OSA, leakage of bacterial RNA and DNA after the accumulation of ROS in the cells	[137]
Gold-aryl NPs	C_6_H_4_-4-COOH linkage in gold	Bovine serum albumin	Internalization in the OSA cells	[138]
Mesoporous silica NPs	Poly acrylic acid, lectin	Doxorubicin	8-folds higher cytotoxicity than free drug	[139]
Micelles	PEG, polyurethane	Doxorubicin	Significant antitumor activity against Saos-2 cells	[140]
Micelles	Polypeptide (methoxy poly(ethylene glycol)-*block*-poly(*S*-*tert*-butylmercapto-L-cysteine) copolymers)	Doxorubicin	Decreased accumulation in the heart and increased accumulation in the OSA cells, inhibition of metastasis	[141]
Nanotube	PLGA	Caspase-3	Suppress proliferation of OSA cells	[142]
Single walled carbon nanotube	graphene	--	ROS mediated cell killing	[143]
Magnetic NPs	Polyethylenimine, dextran, iron oxide	miR-302b	Magnetic field delivered the NPs to the OSA cells and demonstrated cytotoxic effect	[144]
Photoactive mesenchymal stromal cells loaded with NPs	poly-methyl methacrylate	Human osteosarcoma MG-63 cells	Photodynamic therapy to kill OSA cells	[145]

* distearoyl phosphoethanolamine, polyethylene glycol 2000 (DSPE-PEG(2000), ** D-α-tocopherol polyethylene glycol 2000 succinate (TPGS 2000).

## Data Availability

Not applicable.

## Abbreviations

Apa	Apatinib
CaP	Calcium phosphate
CSCs	Cancer stem cells
CT	Commutated tomography
EphA2	Ephrin alpha 2 receptors
FA	Folic acid
GSCT	Gemstone spectral commutated tomography
MLNs	Metastatic lymph nodes
MMP-2	Matrix metalloproteinase-2
MRI	Magnetic resonance imaging
MTX	Methotrexate
NPs	Nanoparticles
OSA	Osteosarcoma
PAI	Photoacoustic imaging
PEG	Poly ethylene glycol
PET	Positron emission tomography
PLGA	Poly (lactide-co-glycolide)
PMAN	Poly(2-(methylacryloyl)ethylnicotinate)
RGD	Arginine-glycine-aspartic acid
ROS	Reactive oxygen species
siRNA	Small interfering RNA
TAMs	Tumor-associated macrophages
